# Cataleptic Hysteria

**Published:** 1856-06

**Authors:** 


### CATALEPTIC HYSTERIA.

Dr. Ringland communicated to the College of Physicians, in Ireland, April 4th, 1855, the following very curious case of cataleptic hysteria:—

Mrs. ——, an English lady, of literary tastes and sedentary habits, about thirty years of age, and married eight years, had been very delicate from her earliest infancy. During the six years antecedent, and the year immediately subsequent to her marriage, she suffered from most intense headache. Two years prior to her marriage she was under treatment for spinal irritation, as she was informed by her then medical attendant. About this period, too, she voided several portions of tapeworm, and had frequently, both before and after her marriage, passed large quantities of ascarides. She was at all times subject to palpitation of the heart, and had on one or two occasions a slight hysterical fit. She suffers intense pain on touching the last dorsal vertebra, which for some years has projected to about the size of a nut. A sound as loud as the snapping of the fingers is frequently heard proceeding from this locality whenever she is much fatigued, or has been standing for a considerable time; and this sound Dr. Ringland has more than once heard. She also experiences, since her first confinement, great pain on the least pressure being made against the coccyx, which has been slightly dislocated downwards and backwards, and has become anchylosed in that direction.

On the second night after she was married, whilst engaged at prayers, she was suddenly, and without the least premonitory indication, seized with the first of the series of fits about to be described; and this was shortly followed by a second; of a like character. An interval of six months then elapsed without their recurrence; when, however, being much about that length of time pregnant, she was again attacked, and, as on the former occasion, without any premonitory symptom, and whilst in a state of complete mental quiescence, having been previously engaged in calm, unexciting conversation with her husband. The headache from which she had previously suffered was greatly aggravated from this period until after her confinement, and she has described it as though a tight iron cap was violently pressed on the upper half of her head, to which the headache was strictly limited. The fits now returned with but very short intervals, and it was with considerable difficulty her medical attendant prevented a premature confinement.

Some little time after the fits became completely established, she observed that, if she was engaged in conversation immediately antecedent to the access of one, she could not command the words she uttered, although fully aware of what she ought to say, and thus she frequently appeared to give expression to the most absurd ideas, and to opinions which were quite opposed to what she had intended to convey. Often, too, having spoken a portion of a sentence, she terminated it on a subject quite different from that upon which she had commenced her observations, or came to an abrupt close, finding herself totally deprived of further utterance.

Up to, and during her confinement, she had frequent attacks, sometimes so many as thirty in the twenty-four hours, and seldom less than fifteen or twenty. After her confinement, which was easy and natural, they were reduced to two or three in the day, and on very rare occasions one whole day has elapsed without their recurrence. Within the last eighteen months her health in this respect has considerably improved, as, repeatedly, days, frequently weeks, and sometimes even a month, has elapsed without a fit.

The origin of this affection she attributes to excessive fright, produced by witnessing very violent paroxysms of hysteria, almost amounting to insanity, in a female relative, with whom she was on a visit shortly before her marriage.

Fatigue, excitement—whether pleasurable or the reverse—or even music—if loud or prolonged—noise—the slightest start—the least pressure against the painful part of the spine, or against the coccyx, instantly induces a fit; they frequently, however, come on without any apparent exciting cause.

The duration of each fit is very variable; sometimes it lasts only three or four minutes, and sometimes it is prolonged to an hour and a half. Dr. Ringland has witnessed several which lasted from twenty minutes to half an hour each.

She has never had less than two fits when attacked, the second being of much shorter duration than the first, and invariably succeeding it after but a short interval.

She appears to have been obnoxious to the attacks at all times and seasons, in all postures, and under every circumstance. She has been liable to them in summer as well as in winter; has been attacked whilst in bed or at her meals; whilst engaged in reading, writing, or in conversation; whilst standing, walking, or sitting; whilst alone or in the midst of strangers;—frequently with a word half uttered, or a piece of food partially masticated; and more than once has her life been placed in jeopardy by the fit occurring when she was near a fire, or whilst she was engaged in the act of deglutition. The presence or absence of menstruation has no apparent connection with the attacks, has that secretion ever been in the least affected by them; neither does the existence of pregnancy or lactation seem in any respect to influence this strange affection, excepting that the fits have been much more frequent from the moment of impregnation up to the period of quickening, than at any other time.

Instantly, on the access of a fit, she falls backwards or forwards, according to the direction in which her head has been at the moment. Should she, however, have her baby in her arms at the time, she holds it firmly clutched in her hands, which cannot, without considerable violence, be opened; although, when the fit ensues at any other time, her hands, though closed, can be easily opened.

The particulars of this lady's case Dr. Ringland learned from herself some months prior to her then approaching confinement—her fourth—and which took place early in December, 1854. Immediately after the birth of the child, which was mature and healthy, she had one of her customary fits, which was followed by a second, immediately after the expulsion of the placenta. The following is a brief description of the first fit witnessed by Dr. Ringland.

Without any previous indication whatever, she suddenly seemed to faint, and lay in a state of *apparently* total unconsciousness. She, however, was quite aware of every circumstance that occurred around her, and could afterwards detail the conversation which had taken place in the room. Her limbs remained in whatever position they were in at the time of the attack, or in any other to which they were subsequently changed. There was no alteration in the color of her lips, in her complexion, or in the appearance of her skin, which remained of the natural temperature. Her eyelids were closed, but when raised, continued open until closed again. The pupils contracted well on exposure to light. Her pulse was about 100, but very feeble. There were no apparent heavings of the chest nor movements of the nostrils. Repeatedly during the existence of the fit, but more violently towards its close, there were convulsive twitchings of the muscles of the face, spasmodic clenching of the fingers, and forcible supination of the hands on the forearm. There were no convulsive movements of the lower extremities, although such occasionally occurred, as she informed Dr. Ringland, and were always present during the first few months of the existence of the fits.

No restoratives were applied during the fit, as she had previously intimated to Dr. Ringland that the employment of the most simple of these had always produced violent and prolonged hysteric paroxysms, which never presented themselves when interference was not had recourse to.

After the lapse of about five minutes she gave a deep sigh, then opened her eyes, looked about her, and feebly held out her hands. On this signal, which is well understood by her attendants, she was without delay raised into a sitting posture, and after a brief interval of quiet she was perfectly restored.

Had not her attendants, as she informed Dr. Ringland, at once placed her in the erect position, she would have relapsed again and again into the fit. She, too, is so conscious of this necessity, that instantly on the subsidence of the fit she holds out her hands, as described, thereby indicating her desire for the requisite assistance. Should she at this time be handled roughly, or should the tender part of the spine or the coccyx be touched, she at once relapses into the fit.

She is not able until after the relapse of considerable time, and not even then without the greatest effort, to utter a single syllable, the peculiar condition excited throughout the system appearing in her case to attach itself more firmly to the tongue than elsewhere.

After the subsidence of the attack she is greatly distressed with tremors of the whole body, which last sometimes for only a few minutes, but at times continue for several hours.

Dr. Ringland, before concluding, made a brief summary of this singular case, directing attention to its leading characteristics and points of interest; especially to the previous existence of spinal irritation; the occurrence of the attacks in summer as well as in winter; the existence of consciousness during the fits; the erect position being necessary at the close of the fit, and neglect in this respect causing relapse; the loss of speech being prolonged after the subsidence of the other symptoms; and finally, to the fact that restoratives induced hysteria.—[*Dublin Quarterly Jour. Med. Sci.*

